# Supplementary material for: Significant Enhancement of the Capacity and Cycling Stability of Lithium-Rich Manganese-Based Layered Cathode Materials via Molybdenum Surface Modification
Source: Molecules. 2022 Mar 24;27(7):2100. doi: 10.3390/molecules27072100 (PMC9000274; doi:10.3390/molecules27072100)
Supplement: Supplementary file 1 [file molecules-27-02100-s001.zip › molecules-1597637-supplementary.pdf]

molecules

MDPI

Supplementary Materials

Significant Enhancement of the Capacity and Cycling Stability of Lithium-Rich Manganese-Based Layered Cathode Materials via Molybdenum Surface Modification

Yijia Shao †, Zhiyuan Lu †, Luoqian Li, Yanni Liu, Lijun Yang, Ting Shu, Xiuhua Li and Shijun Liao *

The Key Laboratory of Fuel Cell Technology of Guangdong Province, School of Chemistry and Chemical Engineering, South China University of Technology, Guangzhou 510641, China; ceyjshao@mail.scut.edu.cn (Y.S.); 15625159545@163.com (Z.L.); 202020123782@mail.scut.edu.cn (L.L.); liuyanniscut@163.com (Y.L.); yanglijun028@gmail.com (L.Y.); shuting6418@163.com (T.S.); lixiuhua@scut.edu.cn (X.L.)

* Correspondence: chsjliao@scut.edu.cn; Tel.: +86-20-871-1358

† These authors contributed equally to this work.

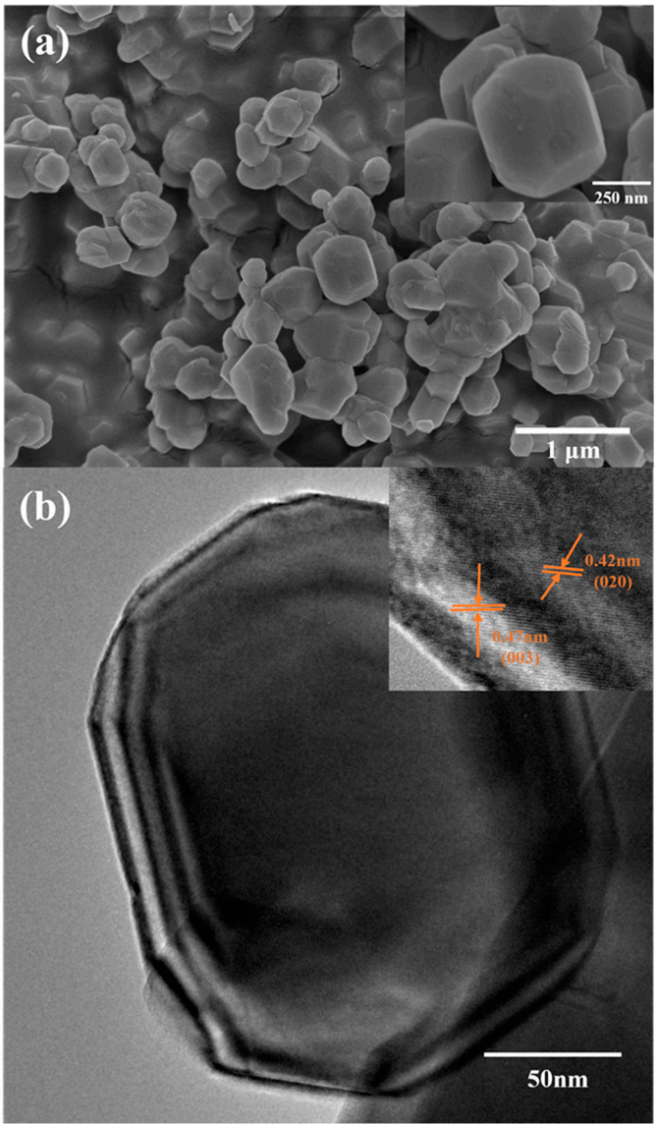

Figure S1. SEM (**a**) and HRTEM (**b**) of LMR sample.

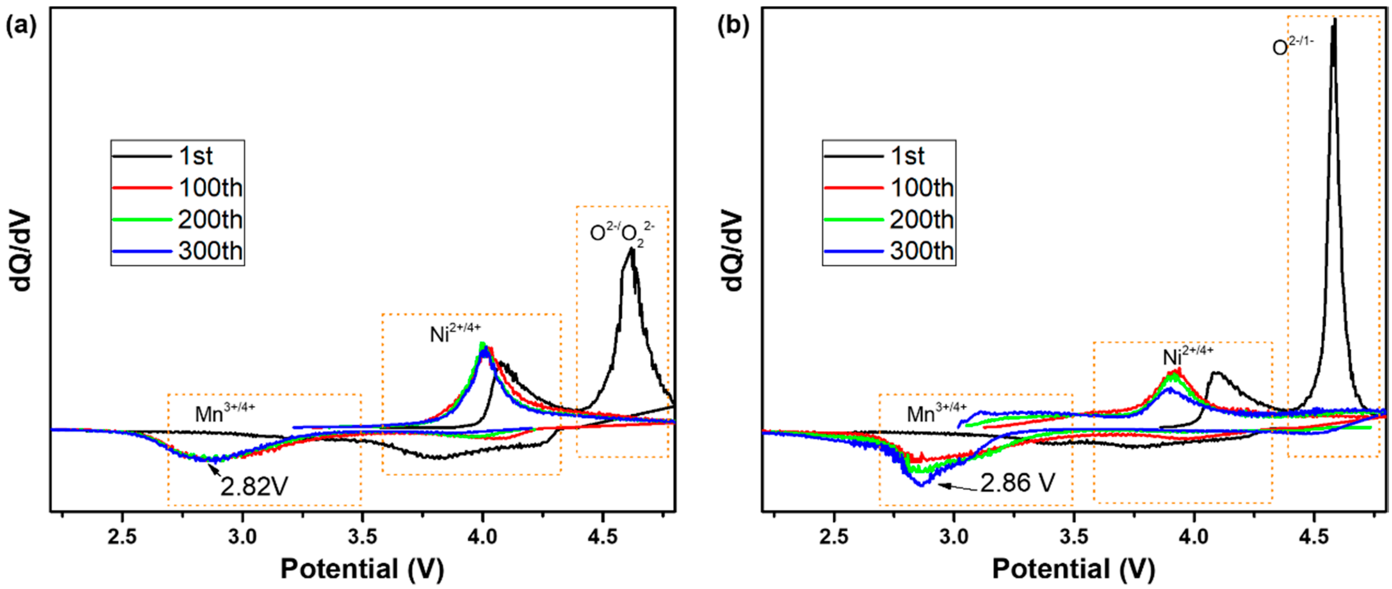

Figure S2. The dQ/dV curves of LMR (**a**) and LMR-Mo (**b**).
